# Evaluation of the Level of Salivary sHLA-G in Children Aged 3–5 Years with or without Dental Caries

**DOI:** 10.1155/2020/8870055

**Published:** 2020-06-30

**Authors:** Mansoureh Bijani, Amrollah Mostafazadeh, Mina Motallebnejad, Ali Bijani, Roghiyeh Pourbagher, Samaneh Gharekhani

**Affiliations:** ^1^Student Research Committee, Babol University of Medical Science, Babol, Iran; ^2^Cellular and Molecular Biology Research Center, Health Research Institute, Babol University of Medical Science, Babol, Iran; ^3^Oral Health Research Center, Health Research Institute, Babol University of Medical Science, Babol, Iran; ^4^Social Determinants of Health Research Center, Health Research Institute, Babol University of Medical Science, Babol, Iran

## Abstract

**Methods and Materials:**

This analytic cross-sectional study was carried out on 83 healthy children aged 3 to 5 years of both genders, who were divided into three groups based on decayed dental surfaces (ds): group 1, caries-free children (CF, *n* = 29); group 2, children with 1 ≤ ds ≤ 3, 1 ≤ ds ≤ 4, and 1 ≤ ds ≤ 5 for age 3, 4, and 5 years, respectively (ECC, *n* = 20); and group 3, children with ds ≥ 4, ds ≥ 5, and ds ≥ 6 for age 3, 4, and 5 years, respectively (S-ECC, *n* = 34). The unstimulated saliva samples were collected, and the salivary sHLA-G concentration was measured by the ELISA kit. The SPSS Statistics v17.0 software and Mann–Whitney, Kruskal–Wallis, chi-square, and Spearman's rank correlation tests were used for statistical analysis. The level of significance was considered at *p* < 0.05.

**Results:**

The mean concentrations of salivary sHLA-G in CF, ECC, and S-ECC groups were 3.18 ± 2.28, 5.64 ± 5.51, and 6.21 ± 6.03 ng/l, respectively (*p* = 0.047), and the mean salivary sHLA-G level was comparatively higher in children with dental caries than that of the CF group (*p* = 0.02), but there is no significant difference between ECC and S-ECC groups (*p* > 0.05). Spearman's rank correlation test showed a weak positive correlation (*p* = 0.039, *r* = 0.22), between the level of salivary sHLA-G and dental caries.

**Conclusion:**

The present study provides some preliminary evidences on relationship between sHLA-G and dental caries in children.

## 1. Introduction

Early childhood caries (ECC) is one of the most common chronic childhood diseases [1]. A review of the literature suggests that in well-developed countries, the prevalence of ECC is between 1 and 12% [2], but in less developed countries, the prevalence has been reported to be as high as 70% [3]. In the north of Iran, the prevalence of S-ECC among 3–6-year-old children was evaluated, highly about 68.8% [4]. This disease has both short-term and long-term effects on children. Specifically, it interferes with their daily activities, as well as their health and developmental capabilities [3, 5]. The American Academy of Pediatric Dentistry (AAPD) defines ECC as the presence of one or more decayed, missing, or filled (due to caries) tooth surfaces (dmfs) in any primary tooth in a child 71 months of age or younger [6]. The dmfs higher than one in a child aged 36 months or younger or in the primary maxillary anterior teeth or dmfs greater than 4, 5, or 6 in any primary tooth for age 3, 4, and 5 years, respectively, constitutes sever ECC (S-ECC) [6]. ECC is a multifactorial infectious disease that results from the interaction of both genetic and environmental factors [7]. The usual risk factors of ECC include poor oral hygiene, diet, low socioeconomic status [8, 9], and host salivary constituents. Dietary practices such as poor dietary habits and food preferences, frequent exposure to sweet beverages, and night-time meals or drinks promote the proliferation of cariogenic bacteria [10, 11]. Inappropriate feeding practices, such as bottle feeding with sweetened milk or fruit juice, night-time bottle feeding, and sleeping with honey-soaked dummies, have been associated with the initiation and development of caries in children [11, 12].

Due to genetic differences such as immunologic response by the host, the susceptibility of individuals to dental caries is different under specific environmental factors. Human leukocyte antigens (HLAs) play a critical role in the immune responses [13, 14]; it is a compilation of genes set on chromosome 6 [15]. Contrasts in HLA molecules can result in differences in the immune response to microorganisms, such as *Streptococcus mutans (S*.*mutans)*, and the susceptibility of teeth to dental caries [16, 17]. HLA-G is a nonclassical HLA class I molecule that differs from classical HLA class I molecule for low allelic polymorphism and restricted tissue distribution [18, 19]. HLA-G molecules are expressed as membrane-bound and soluble isoforms (mHLA-G and sHLA-G), which in turn bind and activate immune-inhibitory receptors such as immunoglobulin (Ig)-like transcript 2 (ILT2) and immunoglobulin (Ig)-like transcript 4 (ILT4), on immune cells [20, 21]. HLA-G molecules are expressing in many tissues including glandular cells [22]. Under normal physiological conditions, the immune cells such as monocytes and dendritic cells (DCs) are the main producers of sHLA-G [23]. The HLA-G molecule expression and release can be increased with different cells such as monocytes and tissues in pathological conditions [13, 24]. The main immune escape mechanism which is used by bacteria is involved in downregulating the immune responses including inhibiting the cytotoxic activity of CD8 positive T lymphocytes (CTLs) and natural killer (NK) cells, inducing the apoptosis of NK and activated cytotoxic T cells, inhibiting antigen-presenting cell (APC) and B lymphocyte differentiation, and inducing regulatory T cells [24]. Since its discovery, many studies described the role of sHLA-G expression in tumor cells and cancers [24–27], recurrent pregnancy loss [28], regulating host immune responses, and recently have begun to be reported in other pathological conditions, including infectious diseases [13, 14]. In particular, HLA-G upregulation has been reported in studies of bacterial infections [29–33]; however, there is no study about the role of salivary sHLA-G in dental caries. This study is the first attempt to evaluate the concentration of salivary sHLA-G and its relationship with dental caries in children.

## 2. Materials and Methods

### 2.1. Study Population

In the present analytical cross-sectional study, 83 children were participated. This sample size was determined based on a previous study [34]. The subjects participated in this study were selected among children aged 3 to 5 years from October 2018 to January 2019 through a convenient sampling method. These children were selected from kindergartens in two cities (Babol and Amol) from north of Iran and the children who were referred to the Pediatric Dentistry Department, Babol Dental School. They were divided into three groups based on the number of decayed tooth surfaces (ds): group 1, caries-free children (CF, *n* = 29); group 2, children with 1 ≤ ds ≤ 3, 1 ≤ ds ≤ 4, and 1 ≤ ds ≤ 5 for age 3, 4, and 5 years, respectively (ECC, *n* = 20); and group 3, children with ds ≥ 4, ds ≥ 5, and ds ≥ 6 for age 3, 4, and 5 years, respectively (S-ECC, *n* = 34). The children suffered from any systemic, allergic, metabolic, and oral disease (gingivitis, acute or chronic dentoalveolar abscess, and acute dental pain) or the ones using antibiotics (since one month before the examination) were excluded from the study. Children with filled or missing teeth were also excluded, and only children with untreated decayed teeth were included in groups 2 and 3.

### 2.2. Data and Sample Collection

Medical history and demographic data of children were obtained using a questionnaire answered by the parents. Clinical examination of all the subjects was carried out by a single trained and calibrated examiner using a sterile mouth mirror and mouth probes under natural light. Subjects were informed not to eat or drink one hour before saliva collection to minimize possible food debris and stimulation of saliva. The unstimulated whole saliva was collected from each subject into a 15 cc sterile falcon tube. Sample collection was conducted between 8 A.M. and 11 A.M., and in less than 2 hours, they were transported in an ice pack to the laboratory and centrifuged at 400 g at 4°C for 10 min and were kept at −80°C until the experiment day.

### 2.3. Laboratory Experiments

The samples were then defrosted and centrifuged for 10 minutes at 400 g at 4°C, and the supernatant was examined by ELISA. The salivary sHLA-G levels were measured by using a HLA-G kit (Shanghai Crystal Day Biotech Co., Ltd., China). The intra-assay coefficient of variation (CV) was lower than 10%, and the interassay CV was lower than 12%. The limit of sensitivity was 2.52 ng/l, and the salivary sHLA-G concentration (ng/l) was determined according to the manufacturer's instructions.

### 2.4. Statistical Analysis

The statistical analysis was performed by SPSS Statistics v17.0 among three groups of children according to dental caries status as the independent variables. The comparisons among the groups were performed according to data distribution. Chi-square, Mann–Whitney, Kruskal–Wallis, and Spearman's rank correlation tests were used for statistical analysis. The level of significance was considered at *p* < 0.05.

### 2.5. Statement of Ethics

The protocol of the study was approved by the Institutional Ethics Committee of the Babol University of Medical Science (IR.MUBABOL.HRI.REC.1397.234). Written informed consent was obtained from all the parents before the study. All children with dental caries have been referred to the Pediatric Dentistry Department of Babol Dental School for treatment of decayed teeth.

## 3. Results

**Figure 1:**
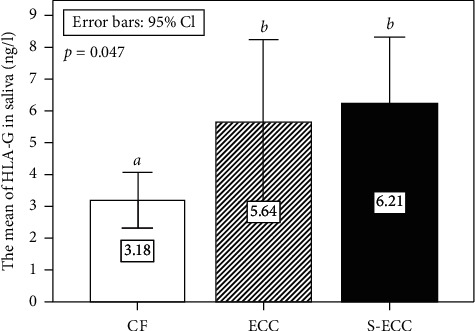
The mean of salivary sHLA-G in each group (std. deviations *a* = 2.28, *b* = 5.51, and *b* = 6.03). There was statistical difference (*p*=0.047) among three groups, considering each HLA-G, separately to the Kruskal–Wallis test (different letters indicate significant differences between groups).

This study was done among 83 subjects (52 girls and 31 boys). The salivary sHLA-G was detected in all samples. The salivary concentration of sHLA-G was significantly different between study groups (Figure 1).

The mean salivary sHLA-G level was comparatively higher in children with dental caries (both ECC and S-ECC groups) than that of the CF group (6.00 ng/l ± 5.80 and 3.18 ng/l ± 2.08, *p*=0.02).We further analyzed the correlation between three group pairs with the Mann–Whitney test. A significant difference was found between ECC and CF as well as S-ECC and CF groups (*p*=0.047 and *p*=0.044, respectively). However, no significant difference between ECC and S-ECC groups is observed (*p* > 0.05). Moreover, the chi-square test suggested an association between salivary sHLA-G and dental caries (Table 1).

Besides, there were no statistical differences among the genders (the chi-square test, *p*=0.31). Spearman's rank correlation test showed a positive correlation (*p*=0.039, *r* = 0.22) between the level of salivary sHLA-G and dental caries.

## 4. Discussion and Conclusions

Since early childhood caries (ECC) is a major disease in the public health, understanding the factors that influence oral health such as biologic factors, parents' behaviours and economy situation [8, 9, 35], data on diet, and preventive behaviour [36] are important for the development of strategies to prevent dental caries and promote oral health. Among biologic factors, the microorganisms, particularly *S*.*mutans*, are one of the main factors in dental caries [37]. So, the immune system and its function against the cariogenic bacteria can play an essential role in causing dental caries. Recently, the sHLA-G is shown to be one of the key elements that affect the immune response. Specifically, it leads to the inhibition of immune responses in many pathological conditions including viral and bacterial infections, tumors, and transplantation [13, 14, 38]. To date, the role of sHLA-G in the maintenance of bacterial infections has been poorly investigated. Some studies showed that during bacterial infections, HLA-G molecules are involved in downregulating the innate immunity [13, 14, 38, 39]. An in vitro study was done by Bortolotti et al. showed that *Pseudomonas aeruginosa* can stimulate HLA-G expression in monocytes and T cells by inducing IL-10 secretion [31]. Additionally, according to the findings of Cao and Mysorekar, HLA-G expression at the cytotrophoblast cell surface increases the risk of *Listeria monocytogenes* infection [40]. Both of these studies highlighted the induction of HLA-G by bacteria to inhibit the host immune system. Since dental caries is also a type of bacterial infection, evaluation of salivary sHLA-G levels can open a new window toward understanding the pathophysiology mechanism in dental caries, especially in children. Inspired by this, in the present study, the levels of salivary sHLA-G and its possible relation with dental caries were evaluated. Specifically, the present study was performed on 83 children aged 3 to 5 years with different severity of dental caries. The results obtained by the present study demonstrate that the concentrations of salivary sHLA-G of children with dental caries (ECC and S-ECC) are significantly higher than the ones without dental caries (CF). However, we were not able to find any statistical significant difference in salivary sHLA-G between subjects with ECC and children with S-ECC groups, where the latter group exhibited higher levels of salivary sHLA-G.

Interestingly, we found a positive association between the concentration of salivary sHLA-G and dental caries (*p*=0.033). We hypothesize the upregulation of sHLA-G secretion caused by dental caries as the main reason of this correlation. Specifically, the antigens such as components of bacteria can stimulate the secretion of cytokines (including IL-10 and IFN-*γ*) by the means of immune cells [41, 42].These cytokines upregulate the expression or secretion of HLA-G [43–45]. The initial protective responses to caries increase the intrapulp pressure and the outward flow of dentinal fluid [42]. The composition of dentinal fluid is not fully determined, but it is considered to be serum-derived tissue fluid containing serum immunoglobulins and its proteins, including sHLA-G [46]. Thus, as the Ig concentration is increased in the saliva of patients with dental caries [47], the level of salivary sHLA-G may also be higher in for patients with dental caries than the ones without dental caries. Additionally immune cells in dentin-pulp interfaces have been found to be inhibited by HLA-G molecules [20, 21]. Recently, it has proven that the antigen-presenting cells (APCs) such as dendritic cells (DCs), have an essential role in the initiation of immune responses [42, 48]. The sHLA-G is the main ligand for the ILT2 and ILT4 receptors which are expressed on DC surface. The interaction of salivary sHLA-G with these receptors leads to the inhibition of maturation and DC activities [20, 21]. When the high levels of salivary sHLA-G pass through dentinal tubules to the pulp, a larger number of DC will be inhibited. Therefore, this mechanism can potentially contribute to the progress of dental caries. We need other studies with larger sample size to evaluate the difference in salivary sHLA-G between ECC and S-ECC groups. All in all, the current study provides some preliminary understanding showing that salivary sHLA-G plays some pathological roles in dental caries. Each person has his own caries risk which is determined by the oral microbiome and immune system. In the future, the concentration of sHLA-G can be potentially used as a biomarker for the early diagnosis of caries and periodontal disease, risk assessment, and individualized caries prevention plan, through the easy-to-access saliva testing technologies such as lateral flow immunochromatographic assay [49, 50]. The lateral flow immunochromatographic assays are used for home or point-of-care testing to detect the presence (or absence) of a target analytic in a sample [51]. This is specifically applicable for early diagnosis of caries in children, where in-home care is preferable and can significantly reduce the cost of healthcare systems. In addition, it requires further study to investigate the cause and effect relationship between two variables. As the samples with acute pulp and gingival inflammation were excluded in this study and particularly due to the anti-inflammatory effects of HLA-G [14]; further research studies can be foreseen on subjects with pulp and gingival inflammation.

## Figures and Tables

**Table 1:** Comparison of salivary sHLA-G level between caries-free children and children with dental caries (the chi-squared test).

Group	The mean of sHLA-G (ng/L)			
	<4	≥4	Total	*p* value
Caries-free children	21	8	29	0.033
Children with dental caries	26	28	54	
Total	47	36	83	

## Data Availability

The patient data used to support the findings of this study are restricted by the Institutional Ethics Committee of the Babol University of Medical Science in order to protect the patient privacy. Data are available from Mansoureh Bijani (mansa.bijani@gmail.com), for researchers who meet the criteria for access to confidential data.
